# Unveiling the status of alien animals in the arid zone of Asia

**DOI:** 10.7717/peerj.1545

**Published:** 2016-01-12

**Authors:** Lyubing Zhang, Zhigang Jiang

**Affiliations:** 1 Key Laboratory of Animal Ecology and Conservation Biology, Institute of Zoology, Chinese Academy of Sciences, Beijing, China; 2 University of Chinese Academy of Sciences, Beijing, China; 3 College of Resources and Environment Science, Xinjiang University, Urumqi, Xinjiang, China

**Keywords:** Alien species, Distribution patterns, Arid zone, Central Asia, Temporal trends, Oasis, Biological invasions

## Abstract

Biological invasion is one of the most threatening factors for biodiversity conservation. Lacking information on alien species in certain regions of the world hampers a balanced understanding of invasion processes and efficient data exchange among stakeholders. Current knowledge gaps are in need of urgent concern. We therefore conducted a review on alien animals in Xinjiang, an unknown region of invasion ecology. Xinjiang lies in the heartland of the Asian continent, covering an area of 1,664,900 km^2^. In the past 64 years, 128 alien animal species were recorded in this region, 39% of which became invasive and led to loss of native biodiversity. Most of these species were introduced through diversification of local agriculture and aquaculture. This process was aggravated by improving transportation and flourishing trade. Multiple linear regression models and correlation analysis were run for explaining influence of environmental and anthropogenic factors on status of alien animals: economically developed areas with abundant water resource, oases in particular, were prone to be hotspots of alien animal species in this arid and semi-arid region. This study also revealed that taxonomically biased and lagged research were critical problems that impeded studies on biological invasions in Xinjiang, and proposed feasible solutions.

## Introduction

Biological invasions caused by alien species (non-native or exotic species) have challenged biodiversity conservation and environmental protection globally (Millennium Ecosystem Assessment, 2005). For coping with present impacts and potential threats, a promising way forward would be to increase effective knowledge transfer among scientists, practitioners and policymakers, in order to facilitate engagement of stakeholders and develop innovative solutions (Hulme, 2015). However, disproportionate research effort in different regions has hindered a balanced understanding on biological invasions. Intensive research in specific regions, such as Europe and North America, does provide in-depth knowledge to this filed (Wilson et al., 2007). Nevertheless, complex and context-dependent processes in generalization of invasion processes are likely to be impeded in certain parts of the world, especially in Asia and tropical Africa, by scarce availability of studies (Crall et al., 2006; Pyšek et al., 2008). Asia is a continent with dense human activities and high habitat heterogeneity, holding the lowest number of naturalized alien species (Pyšek et al., 2008). Such status may be the result of a combination of both insufficient research and incomplete recording caused by under-represented information of alien species in English-language literature (Su, Cassey & Blackburn, 2014). For helping filling these gaps of knowledge, it is necessary to collect data from those “poorly-known regions” of biological invasions and deliver them to the globe.

In this study we conducted a review related to alien animal species occurring in China’s Xinjiang Uyghur Autonomous Region (N 34°22′–49°33′, E 73°41′–96°18′, Xinjiang hereafter), for contributing to fill in the information gaps of biological invasions in Asia. Xinjiang is a region spanning 1,664,900 km^2^ located in the heartland of the Asia Continent, encompassing the geographical centre of Asia (N 43°40′37″, E 87°19′52″). Divided by the centrally located Tianshan Mountain Range, Xinjiang has two main geographic regions: north and south. The northern region of Xinjiang mainly contains the Junggar Basin, the Ili Valley and south part of the Altay Mountains. The southern region includes Kunlun Mountains, East Pamirs, the Tarim Basin which is dominated by Taklimakan Desert, and oases around the basin. Most of areas in Xinjiang are arid or semi-arid because of the strong influence of continental climate. To increase farmlands, local government built channels to divert water from rivers and lakes for extending oases into the desert, thereby facilitating the formation of semi-artificial landscapes. Zoogeographically, Xinjiang belongs to the Palearctic realm, bordering the Sino-Japanese realm in the south (Holt et al., 2013). Its native fauna is mainly composed of desert animal group, alpine desert group and alpine forest steppe group (Zhang, 1999).

Xinjiang deserves attention of invasion ecology not only for its geographic location but also for its economic context. During ancient times, the famous trade route “Silk Road,” which linked the city Chang’ an (located near to the modern city Xi’an in northwest China) and the Mediterranean Sea region, had passed through Xinjiang since the Han Dynasty (206 B.C.–220 A.D.). However, the region was in economic isolation when the trading along the Silk Road vanished at the fall of the Mongol Empire. Since the middle of the 20^th^ century, powered by economic and transportation development of China (Ding et al., 2008; Wu et al., 2010), Xinjiang has reopened to the world at unprecedented pace and scale. In 2014, in order to revive the ancient trade route, the Chinese government unveiled the blueprint of the “New Silk Road”: Xinjiang will become the forefront of national economic development, which implies a faster increase of human activities in the future. In this complex ecological and socioeconomic context, we intended to answer the following questions related to Xinjiang’s alien animals, as well as the causes and consequences on the region: what alien animal species have been detected in Xinjiang? How did they arrive here? What are the main factors driving such a process? What are their biological and social impacts? By answering these questions, the study strives to provide clearly and openly regional information to global research, and will also help to promote local studies by revealing existing threats, for the sake of developing effective management strategies on biological invasions.

Supported by experts with taxonomic and ecological expertise, we compiled data on alien animal species in Xinjiang from both English and Chinese literature. Specifically, this study aimed to: (1) present an updated inventory of alien animal species in Xinjiang; (2) assess taxonomic composition of listed alien animal species, as well as characterize variations in their distribution and temporal trends, and (3) explore environmental and anthropogenic factors associated with the arrival, establishment and invasion of alien animals in the context of an arid and semi-arid region.

## Methods

### Data extraction

Most of the records of alien animal species in Xinjiang were extracted from the “Biodiversity Assessment Report of Xinjiang” (Yuan, Li & Lv, 2012) which contributed with its data to the Biodiversity Assessment Project of China. However, some alien animals were missing in this report as a result of incomplete knowledge on definition of “alien species.” Therefore, we followed the definition of alien species proposed by Richardson, Pyšek & Carlton (2011), as “species have been moved beyond their native geographic range by human activity.” Records of animal species were then retrieved in *Fishes of Xinjiang Uygur Autonomous Region, China* (Guo, Zhang & Cai, 2012b), *A checklist on the distribution of the birds in Xinjiang* (Ma, 2011), and the *Handbook of insects in Xinjiang* (Hu et al., 2013) to identify alien species according to the definition. Native or global distribution range of these species were recognized using the range descriptions in the Catalogue of Life (Roskov et al., 2015), Global Invasive Species Database (ISSG, 2014), and the Invasive Species Compendium (CABI, 2014). For the species’ native range described as “China” in these databases, we specified their native range on national scale according to the species range map developed by the IUCN Red List (IUCN, 2014) and descriptive records in the *Fauna Sinica* (CAS, 2015). Information on introduction history and population status was extracted from peer-reviewed literatures. Considering that many papers were published in Chinese, our literature search was performed in the China Integrated Knowledge Resources Database (China Academic Journals Electronic Publishing House Co. Ltd., 2014) to access data those are not covered by the online search engine Web of Science™. We searched all available fields including the title, abstract, article, topic, and full text containing the terms: “alien,” “introduced,” “non-native” or “exotic species” or “biological invasions” or alien species names (such as *Ondatra zibethicus*), and “Xinjiang” to extract information. Information from grey literatures was referenced provided that reliability was confirmed through further expert consultation.

### Data compiling and analysis

We compiled a dataset organized by taxonomic groups, containing the records of occurrence site, population status, native range, likely introducing pathways, and first recorded time in Xinjiang. Population status of the alien animal species was defined by current population size and changes in range size, according to the definition in the unified framework for biological invasions (Blackburn et al., 2011): “non-established population”: only a few live individuals in a single location but either failed to survive or fail to reproduce, or propagated several individuals within the barrier of captivity of cultivation. “Established population” refers to established self-sustaining populations in wild after several generations. “Invasive population” referes to a population with individuals disperse, survive and reproduce in a range with significant distance from the original point of introduction. Species that lack of population data or had no relevant description were classified as “present but no details.” Likely pathways of introductions were classified as aquaculture, farming, pet trade, unintentional introductions along with plants or aquatic products. The year when the alien animal species was recorded for the first time in Xinjiang was also included in the dataset if the information was available. We performed the following analysis on the alien species with the dataset.

To explore influencing factors of alien animal invasions, we selected 10 potential explanatory variables and divided them into three classes related to environmental (I–II) and anthropogenic factors (III) as follows: (I) Geography: (1) total land area (km^2^); (2) total area of wetlands, including area of rivers, lakes and marshland (km^2^); (3) annual volume of surface water resource (ton). (II) Climate: (4) mean annual temperature (°C); (5) mean annual precipitation (mm); (6) difference in mean July and January temperature (°C). (III) Anthropogenic factors: (7) land use, area of land used by human in the prefecture (km^2^), including land for farm use such as cultivation, gardening, woodland, pastureland *etc.*, and land for construction; (8) gross domestic production (GDP) of each prefecture (USD); (9) transportation development status, using the share of transportation output in GDP as the surrogate (%); (10) status of foreign trade development, reflected by the share of imports in GDP (%). We extracted values of all these variables for each prefecture (an administrative level under province but above county in China) from the *Statistic Yearbook of Xinjiang* (Chen, 2014). Statistic data after 1978, the year when systematic statistics began in Xinjiang, were used for analysis despite of the limitation on explaining historical processes of invasion (Pyšek et al., 2010). Multiple linear regression models were used to account for the relationship between potential variables and the number of alien species of each prefecture. To normalize the data, prefecture area, wetland area, area of land used by human, GDP of each prefecture, transportation share of GDP and imports share of GDP were log-transformed before analysis. Durbin-Watson test was used for assessing the independence of the residuals (explained in detail in Table S1). Collinearity of variables was checked by calculating tolerance values, eigenvalues and condition indices. We conducted correlation analysis to characterize temporal associations between number of new records of alien animal species per year and anthropogenic factors. We averaged GDP, transportation share of GDP and import share of GDP of every five-year period for analysis. We excluded environmental factors and land used by human in this part of analyses because they were relatively constant on a 30-year temporal scale.

## Results

### Alien animal species in Xinjiang

**Figure 1:**
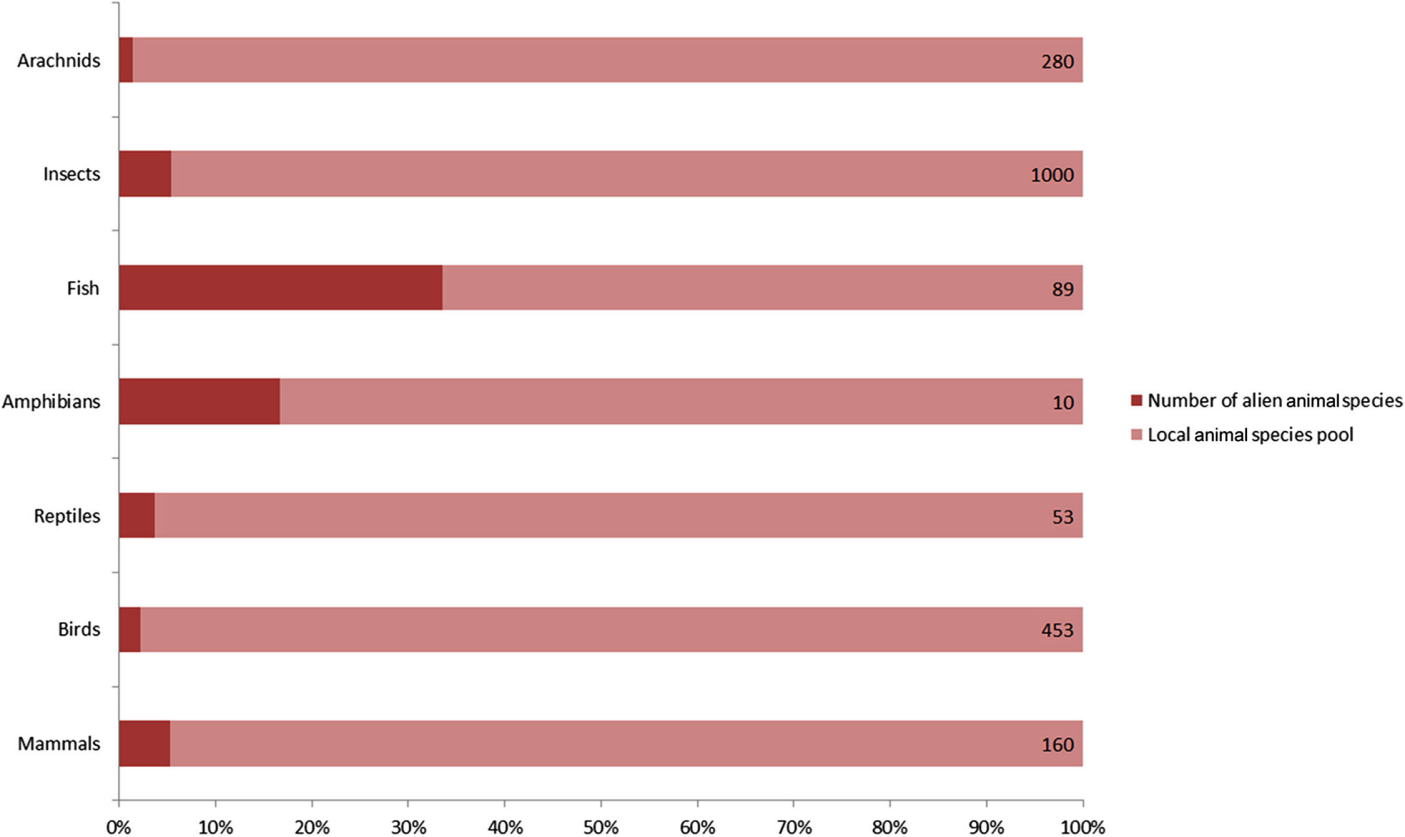
Number of recorded alien animal species and aliens’ ratio in local animal species pool. Capacity of local animal species pool for each taxon shown on the right end of the bar was extracted from several peer-reviewed publications on fauna of Xinjiang. Arachnids: Hu & Wu (1989) (Supplemental Reference); Birds: Ma (2011); Mammals, reptiles and amphibians: Yuan, Li & Lv (2012); Fishes: Guo, Zhang & Cai (2012b); Insects: Hu et al. (2013).

A total of 128 alien animal species have been recorded in Xinjiang since 1950, including 9 mammals, 9 birds, 2 reptiles, 2 amphibians, 45 fishes, 57 insects and 4 arachnids (Table 1). They accounted for 9% of 765 vertebrates, and nearly 5% of over 1280 insects and arachnids of local fauna (Fig. 1). According to the definition of invasion stages (Blackburn et al., 2011) and status of the alien populations, we determined that 40 of 128 species have already established self-sustaining populations in the wild. 50 species were determined as “invasive” for their massive population growth and range expanding observed, which constituted nearly a quarter of 198 invasive alien animal species recorded in China (Xu et al., 2012). For these invaders in Xinjiang, terrestrial arthropods accounted for the majority (36 species, 72%), followed by fishes (11 species, 22%). A total of 16 species were determined as cultivated populations kept within man-made barriers and 22 species were recorded as “present, without details.” Referring to the ‘10s rule’ (10% of alien species can establish self-sustaining population, and 10% of these established species can become invasive or problematic), the rate of established and invasive species in Xinjiang appears to be exceptionally high. A possible reason is that the actual number of alien animal species should be much higher than the previously recorded, because it was often hard to detect species less impacting local economy or biodiversity (non-problematic species). A total of 67% of recorded vertebrate alien animals and a honey bee (*Apis mellifera*) were intentionally introduced. Unintentional introductions included following two cases: alien animal species that were brought into Xinjiang with commodities (79% of listed arthropods and 31% of fishes), and alien animal species that had been introduced to neighboring areas (for example, Kazakhstan) by human before they dispersed into Xinjiang naturally (12 species). As to likely pathways of introduction, five in nine alien mammal species were introduced for fur farming, eight of nine alien bird species were traded into Xinjiang as pets, 87% of alien fishes were introduced for aquaculture, 75% alien insects and all alien arachnids were carried to Xinjiang along with flows of agricultural products, plantlets, fruits or ornamental flowers. Chinese softshell turtle (*Pelodiscus sinensis*), wattle-necked softshell turtle (*Palea steindachneri*) and common bullfrog (*Lithobates catesbeianus*) were introduced for aquaculture. Common clawed frog (*Xenopus laevis*) entered Xinjiang as pets. Detailed information on alien animals of Xinjiang was listed in Table 1.

**Table 1:** List of alien animal species recorded in Xinjiang.

Order	Species	Population Status^a^	1st Recorded Time^b^	Introducing Pathways^c^	Native Range^d^	Distribution Range^d^
**Artiodactyla**						
	*Cervus nippon* (Temminck, 1838)	NE	2010	If	Southern China; Japan; Russian Federation	Australia; Europe & Northern Asia (excluding China); North America; Southern Asia
	*Lama glama* (Linnaeus, 1758)	NE	2004	If	South America	
**Carnivora**						
	*Neovison vison* (Schreber, 1777)	E	1963	If	North America	North America; Europe; Japan
	*Mustela lutreola* (Linnaeus, 1761)	E	1963	If	France; Romania; Russian Federation; Spain; Ukraine	Europe and Northern Asia (excluding China)
	*Urocyon cinereoargenteus* (Schreber, 1775)	P	1997	If	Middle America; North America; South America	Middle America; North America; South America
	*Nyctereutes procyonoides* (Gray, 1834)	NE		If	Far East Asia; Eastern and Southern China; Japan	Europe and Northern Asia; Southern Asia
**Rodentia**						
	*Ondatra zibethicus* (Linnaeus, 1766)	I	1950s	ND+If		Europe and Northern Asia (excluding China); North America; South America; Southern Asia
	*Rattus norvegicus* (Berkenhout, 1769)	I	1975	UI		Australia; North America; Southern Asia
	*Rattus flavipectus* (Milne-Edwards, 1871)	E	1994	UI	Shanxi, Gansu provinces and most southern areas of China^1^	
**Anseriformes**						
	*Branta canadensis* (Linnaeus, 1758)	P	after 2001	U	Middle and North America	Middle America; North America; Oceania; Europe
**Psittaciformes**						
	*Trichoglossus haematodus* (Linnaeus, 1771)	P	after 2001	Ipe	Indonesia; New Caledonia; Papua New Guinea; Solomon Islands; Vanuatu	
	*Psittacula krameri* (Scopoli, 1769)	P		Ipe	Central Africa; South Asia	Southern and western Asia; Europe; Central Africa; North America; Oceania
**Passeriformes**						
	*Sturnus nigricollis* (Paykull, 1807)	P	before 2010	Ipe	Southeastern Asia	
	*Spodiopsar sericeus* (Gmelin, 1789)	P	2011	Ipe	Southeastern China^2^	
	*Acridotheres cristatellus* (Linnaeus, 1758)	P	before 2010	Ipe	Southern China; Lao; Myanmar; Taiwan; Vietnam	
	*Acridotheres tristis* (Linnaeus, 1766)	I	1986	Ipe+ND	Southern and western Asia	
	*Spodiopsar cineraceus* (Temminck, 1835)	P	2006	Ipe	Eastern and central China^2^; Mongolia; Japan; Korean Peninsula	
	*Lonchura oryzivora* (Linnaeus, 1758)	P		Ipe		Caribbean; Oceania; Southern Asia
**Anura**						
	*Lithobates catesbeianus* (Shaw, 1802)	E	1967	Ia	North America	North and central America; Southwestern Europe; Japan; China
	*Xenopus laevis* (Daudin, 1802)	P		Ipe	Central and southern Africa	
**Testudines**						
	*Pelodiscus sinensis* (Wiegmann, 1835)	NE		Ia	Central and Southern China; Lao’s; Japan	
	*Palea steindachneri* (Siebenrock, 1906)	NE		Ia	Southern China; Lao’s; Vietnam	
**Acipenseriformes**						
	*Acipenser nudiventris* (Lovetsky, 1828)	E	1945	Ia+ND		Black, Azov, Caspian and Aral Sea
**Salmoniformes**						
	*Oncorhynchus mykiss* (Walbaum, 1792)	E	1996	Ia	Native to Pacific Slope from Kuskokwim River, Alaska to (at least) Rio Santa Domingo, Baja California, Mexico; upper Mackenzie River drainage (Arctic Basin), Alberta and British Columbia in Canada; endorheic basins of southern Oregon, USA	
	*Oncorhynchus aguabonita* (Jordan, 1892)	P		Ia		North America
	*Oncorhynchus masou* (Brevoort, 1856)	NE	2006	Ia		Kamchatka, Kuril Islands, Sakhalin, Primorsky Krai south through Korea, Taiwan and Japan
	*Salvelinus malma* (Walbaum, 1792)	NE	2004	Ia		Arctic, Northwest to Northeast Pacific
	*Coregonus peled* (Gmelin, 1789)	I	1998	Ia		Lakes and rivers from Mezen to Kolyma River, Russia
	*Coregonus migratorius* (Georgi, 1775)	E	1998	Ia		Lake Baikal in northern Siberia. Also enters the tributaries Kichera, Verkhnyaya Anagara, Chivyrkui, Barguzin and Selenga
**Osmeriformes**						
	*Hypomesus nipponensis* (McAllister, 1963)	I	1989	Ia	Northwest Pacific	Japan to the Korean Peninsula
	*Protosalanx hyalocranius* (Abbott, 1901)	E	1995	Ia		Northwest Pacific: Korea and temperate coast of China
**Cypriniformes**						
	*Cyprinus carpio* (Linnaeus, 1758)	I	1905	Ia		Black, Caspian and Aral Sea basins
	*Pseudorasbora parva* (Temminck & Schlegel, 1846)	I	1980	UIa		Amur to Zhujiang drainages (Siberia, Korea and eastern China)
	*Abramis brama* (Linnaeus, 1758)	I	1959	UIa+ND		Most European drainages from Adour (France) to Pechora (White Sea basin); from Marmara basin (Turkey) and eastward to Aral basin; Introduced in Lake Baikal and upper Ob and Yenisei drainages
	*Ctenopharyngodon idella* (Valenciennes, 1844)	E	1963	Ia		Northeastern China to eastern Siberia (Amur River system)
	*Hypophthalmichthys molitrix* (Valenciennes, 1844)	I	1959	Ia	Major Pacific drainages of East Asia (from Amur to southeastern China)	
	*Hypophthalmichthys nobilis* (Richardson, 1845)	E	1950s	Ia	Eastern China^3^	
	*Carassius auratus* (Linnaeus, 1758)	I	1960	UIa	Main drainages in eastern China, Taiwan, Korean Peninsula	
	*Ictiobus cyprinellus* (Valenciennes, 1844)	NE	2000	Ia	North America	
	*Mylopharyngodon piceus* (Richardson, 1846)	NE		Ia		Amur river basin to southern China of Asia
	*Leuciscus aspius* (Linnaeus, 1758)	I	1958	Ia		European drainages; Introduced in Rhine, Northern Dvina and Lake Balkhash (Asia)
	*Parabramis pekinensis* (Basilewsky, 1855)	NE		Ia		Basin of the Amur, from Blagoveshchensk to the very mouths. Sungari, Ussuri, Lake Khanka, Liao. China south to Shanghai and Ningpo
	*Hemiculter leucisculus* (Basilewsky, 1855)	E		UIa		Eastern China, North and South Korea, Hong Kong, Japan and Amur River basin to Red River drainages; Mongolia. Introduced in Iran
	*Misgurnus mohoity* (Dybowski, 1869)	E		UIa		Northeast rivers (Heilongjiang) of China^3^; Mongolia and Russia
	*Abbottina rivularis* (Basilewsky, 1855)	I	1998	UIa	Eastern China	Eastern China, Korea and Japan. Introduced in the Mekong Basin. Recorded from Tedzhen River basin in Turkmenistan
	*Hemibarbus maculatus* (Bleeker, 1871)	E	1960	UIa		Eastern China^3^, Korea, Japan and Amur River basin
	*Megalobrama amblycephala* (Yih, 1955)	NE	1980	Ia		Middle reaches of Yangtze River, mainland China.
	*Megalobrama terminalis* (Richardson, 1846)	P	1965	UIa		Amur basin to southern China
	*Rhodeus ocellatus* (Kner, 1866)	E		UIa		Eastern Asia and Taiwan; Russia
	*Rhodeus sinensis* (Günther, 1868)	E		UIa		South of Yangtze River, China; Korea
	*Luciobarbus brachycephalus* (Kessler, 1872)	E	1996	Ia+ND		Southern and western Caspian Sea; Aral basin; River Chun (Kasakhstan)
	*Leuciscus baicalensis* (Dybowski, 1874)	I	1964	Ia+ND		Ulungur Lake and Ulungur River in China (introduced^4^); Mongolia and rivers draining to the Arctic Ocean, from the Ob to the Kolyma
**Siluriformes**						
	*Silurus meridionalis* (Chen, 1977)	E	1991	Ia		Middle Yangtze River basin, China^3^
	*Silurus soldatovi* (Nikolskii & Soin, 1948)	E	1970	Ia+ND		Amur basin (Asia)
	*Clarias fuscus* (Lacepède, 1803)	NE	1990	Ia		Subtropical area of China; Taiwan; Philippines and Vietnam; Hawaii
	*Clarias camerunensis* (Lönnberg, 1895)	NE		Ia		Coastal rivers in Togo to the lower and middle Congo River basin
	*Ameiurus nebulosus* (Lesueur, 1819)	E	1989	Ia		North America drainages; Iran and Turkey
	*Ictalurus punctatus* (Rafinesque, 1818)	NE	1990	Ia		North America: Central drainages of the United States to southern Canada and northern Mexico
**Beloniformes**						
	*Oryzias sinensis* (Chen, Uwa & Chu, 1989)	E		UIa		Mekong, Irrawaddy, Salween, Red River and Nanpangjiang basins. Introduced in Kazakhstan where it is now abundant; established in lower Kuban drainage; spreading in Azov basin, discovered in River Obitochnaya, Ukraine
**Perciformes**						
	*Sander lucioperca* (Linnaeus, 1758)	E	1965	Ia+ND		Caspian, Baltic, Black and Aral Sea basins; Elbe (North Sea basin) and Maritza (Aegean basin) drainages. North to about 65° N in Finland. Introduced widely
	*Siniperca chuatsi* (Basilewsky, 1855)	NE	1990	Ia		Amur River basin and China
	*Oreochromis niloticus* (Linnaeus, 1758)	NE	1983	Ia		Africa. Widely introduced for aquaculture
	*Rhinogobius cliffordpopei* (Nichols, 1925)	E	1998	UIa		South China^3^ and Vietnam
	*Rhinogobius brunneus* (Temminck & Schlege, 1845)	E	1998	UIa		River basin of the seas of Japan; Taiwan, rivers of Korea; South of Yangtze River and Nanpanjiang River of China^3^; Philippines; Vietnam. Introduced to the USA
	*Macropodus opercularis* (Linnaeus, 1758)	E	2005	UIa		China, from Yangtze basin to the south, on Hainan Island, in Taiwan, north Vietnam; introduced to the tropical and subtropical world
	*Channa argus* (Cantor, 1842)	E	2000	Ia		Amur southward to Xi Jiang and Hainan Island, China; Introduced elsewhere. Japan and the USA
	*Micropercops cinctus* (Dabry de Thiersant, 1872)	I		UIa		Amur southward to Xi Jiang, China
**Prostigmata**						
	*Tetranychus urticae* (Koch, 1836)	I		UIpl	US; Western Europe and Mediterranean	Worldwide
	*Amphitetranychus viennensis* (Zacher, 1920)	I	1981	UIpl		
	*Panonychus ulmi* (Koch, 1836)	I	1995	UIpl	Europe	
	*Aceria macrodonis* (Keifer, 1965)	E	1991	UIpl		
**Blattodea**						
	*Blattella germanica* (Linnaeus, 1767)	I	1980	UI+ND		Worldwide, African origin
**Hymenoptera**						
	*Bruchophagus gibbus* (Boheman, 1836)	I		UI		European origin
	*Apis mellifera* (Linnaeus, 1758)	I	1900	If		Africa; Africa; Australia; Caribbean; Europe & Northern Asia (excluding China); Middle America; North America; Oceania; South America; Southern Asia
	*Urocerus gigas* (Linnaeus, 1758)	P	1984	U		Europe, North America
	*Janus piri* (Okamoto & Muramatsu, 1925)	I	1987	UIpl		Mainland China (exclude western China), Korean peninsula^1^
**Lepidoptera**						
	*Anarsia lineatella* (Zeller, 1839)	P		UIpl	Mediterranean Sea	
	*Cydia pomonella* (Linnaeus, 1758)	I	1950	UIpl+ND		Europe^5^
	*Grapholita inopinata* (Heinrich, 1928)	P		UIpl		Korean Peninsula; Japan; the Russian Far East^1^
	*Carposina sasakii* (Matsumura, 1900)	P		UIpl		Japan; Korean Peninsula^5^
	*Pieris rapae* (Linnaeus, 1758)	I		UIpl		North America; Europe; Japan; Oceania^5^
	*Paranthrene tabaniformis* (Rottemburg, 1775)	I	1965	UIpl		
	*Aegeria molybdoceps* (Hampson, 1919)	I	1995	UIpl		
	*Opogona sacchari* (Bojer, 1856)	E	1995	UIpl		Sub-Saharan Africa; Mauritius
	*Monema flavescens* (Walker, 1855)	I	2001	UIpl		Korea; Japan; the Russian Far East (Amur, Ussuri, Askold); Eastern and central China^1^
	*Acleris fimbriana* (Thunberg, 1791)	E	2008	UIpl		
**Diptera**						
	*Liriomyza huidobrensis* (Blanchard, 1926)	I	1998	UIpl		California, Utah, Washington, Neotropics, Easter Isl., Hawaii
	*Liriomyza sativae* (Blanchard, 1938)	I	1996	UIpl		US, Guam, Hawaii, New Caledonia; Neotropical areas
	*Mayetiola destructor* (Say, 1817)	E	1975	UIpl		Europe^5^
	*Carpomya vesuviana* (Costa, 1854)	E	2007	UIpl+ND	India	Italy, Bosnia, Caucasus, Central Asia, Pakistan, India, Thailand
	*Meromyza saltatrix* (Linnaeus, 1761)	P		UIpl	Europe^5^	Europe, Russia, Mongolia, China; North America
	*Liriomyza bryoniae* (Kaltenbach, 1858)	I	1998	UIpl	Germany	
**Coleoptera**						
	*Araecerus fasciculatus* (DeGeer, 1775)	P		U	India^5^	Worldwide
	*Bruchus pisorum* (Linnaeus, 1758)	I	1965	U	Mediterranean Sea^5^	Canada; US; Europe; Northern Asia
	*Leptinotarsa decemlineata* (Say, 1824)	I	1993	UIpl+ND	US^5^	Canada; Continental US; Europe & Northern Asia
	*Hypera postica* (Dejean)	I		U		European origin
	*Sitophilus granarius* (Csiki, E., 1936)	I	1954	UI	Canada, US	
	*Cryptorhynchus lapathi* (Dejean)	I		UIpl	Canada, US	Europe, Canada, US and Japan
	*Cosmopolites sordidus* (Marshall, G.A.K., 1930)	P		U	Malaysia and Indonesia	
	*Anthrenus picturatus* (Solsky, 1876)	P		U		Europe^5^
	*Anthrenus verbasci* (Linnaeus, 1767)	P		U		Present in most of Europe, in East Palearctic ecozone, in the Near East, in the Nearctic ecozone and in North Africa.
	*Lissorhoptrus oryzophilus* (Kuschel, 1952)	I	2008	UIpl	American continent	
	*Agrilus mali* (Matsumura, 1924)	E	1993	UIpl		Korean Peninsula; Japan^5^
	*Anoplophora glabripennis* (Motschulsky, 1854)	I	1999	UIpl	Eastern China; Japan; Korea	
	*Monohelea hieroglyphica* (Kieffer, 1917)	I	1998	UIpl		Guyana; Brazil; West Indies (Dominica, Trinidad)
	*Potosia brevitarsis* (Lewis)	I	2001	UIpl		Northeastern and central China^6^
	*Apriona swainsoni* (Hope, 1840)	E	2003	UIpl		South China; India; Lao’s, Vietnam
	*Semanotus bifasciatus* (Motschulsky, 1875)	E	2003	UIpl		China; Japan; Mongolia; Russia
	*Chrysobothris succedanea* (Saunders)	E	2008	UIpl		Eastern China, Gansu, Ningxia^6^
**Thysanoptera**						
	*Frankliniella occidentalis* (Pergande, 1895)	I	2007	UIpl	Southwestern United States	Africa; Africa; Australia; Caribbean; Europe & Northern Asia (excluding China); Middle America; North America; Oceania; South America; Southern Asia
**Hemiptera**						
	*Eulecanium giganteum* (Shinji, 1935)	I	1979	UIpl		Russia; China; Japan
	*Phenacoccus solenopsis* (Tinsley, 1898)	I	2010	UIpl		Mexico; Dominican Republic; United States of America; Panama; Ecuador; Cuba
	*Diuraphis noxia*	I	1977	UIpl		
	*Trialeurodes vaporariorum* (Westwood, 1856)	I	1978	UIpl	Europe	
	*Zyginidia eremita* (Zachvatkin, 1953)	I	1982	UIpl		
	*Aphis gossypii gossypii* (Glover, 1877)	I	1984	UIpl		Worldwide
	*Eriosoma lanigerum*	E	2005	UIpl		Originated in eastern North America
	*Aphanostigma piri* (Cholodkovsky, 1904)	E		UIpl		
	*Bemisia tabaci* (Gennadius, 1889)	I	1998	UIpl	India	
	*Pseudaulacaspis pentagona* (Targioni Tozzetti, 1886)	I	1990	UIpl		Originated in eastern Asia
	*Parlatoria oleae* (Colvée, 1880)	I	1994	UIpl		Egypt, Morocco, Spain, Sicily, Saudi Arabia, Sardinia, Romania, Pakistan, Malta, Lebanon, Kazakhstan, United States of America, Sri Lanka, Portugal, Sudan, Syria, Taiwan, Tajikistan (=Tadzhikistan), Tunisia, Turkey, Turkmenistan, Jordan, United Kingdom, Libya, Uzbekistan, Yugoslavia, Ukraine, Argentina, Mexico, Algeria, Italy, Armenia, Australia, Azerbaijan, Belgium, Bolivia, Brazil, Bulgaria, Canary Islands, Cayman Islands, China, Crete, Iraq, Afghanistan, Cyprus, Israel, Iran, India, Hungary, Germany, Georgia, France, Greece, Ethiopia
	*Cacopsylla chinensis* (Yang & Li, 1981)	E	1997	UIpl		
	*Arboridia apicalis* (Nawa, 1913)	I	1999	UIpl		
	*Nipaecoccus nipae* (Maskell, 1893)	E	2001	UIpl		Europe, Asia, Africa, North, Central and South America and Oceania
	*Heliococcus ziziphi* (Borchsenius, 1958)	I	2003	UIpl		China
	*Pseudococcus maritimus* (Ehrhorn, 1900)	E	2003	UIpl		French Guiana, Poland, United States of America, Mexico, Indonesia, Guatemala, Canada, Brazil, Bermuda, Armenia, Argentina, Chile
	*Batracomorphus pandarus* (Knight 1983)	E	2009	UIpl	Malaysia (Sabah, Sarawak)	
	*Coccura suwakoensis* (Kuwana & Toyoda, 1915)	E	2011	UIpl	Japan	Russia; Japan; North Korea; China

**Notes:**

a Population status: *E* established, *NE* non-established, *I* invasive, *P* present but no details.

b First recorded time is the year when a certain alien animal species was recorded or reported for the first time in Xinjiang. Data were extracted from peer-reviewed literatures and local fauna provided in the Supplemental References.

c Introducing pathway: *If* introduction for farming, *Ipe* escaping or released pets, *Ia* introduction for aquaculture, *ND* naturally dispersed to Xinjiang after being introduced, *UI* unintentional introduction, *UIa* unintentional introductions with aquatic products, *UIpl* unintentional introductions with plants (such as crops, ornamentals, fruit trees and timber), *U* unknown pathways.

d We gave distribution and native range of species if information was available in following databases: Catalogue of Life (Roskov et al., 2015), IUCN Redlist (IUCN, 2014), Global Invasive Species Database (ISSG, 2014) or Invasive Species Compendium (CABI, 2014). For those ranges recorded as “China” in global databases, we specified them using:

1 China Animal Scientific Database (CAS, 2015).

2 A field guide to the birds of China (MacKinnon, Philipps & He, 2001).

3 Distribution and zoogeographic division of freshwater fish in China (Li, 1981).

4 Fishes of Xinjiang Uygur Autonomous Region, China (Guo, Zhang & Cai, 2012b).

5 Database of Invasive Alien Species of China (CMIAS, IPP).

6 China Agriculture Pests Information System (IPP). Above references were listed in the references or in the Supplemental References.

### Distribution of the aliens

**Figure 2:**
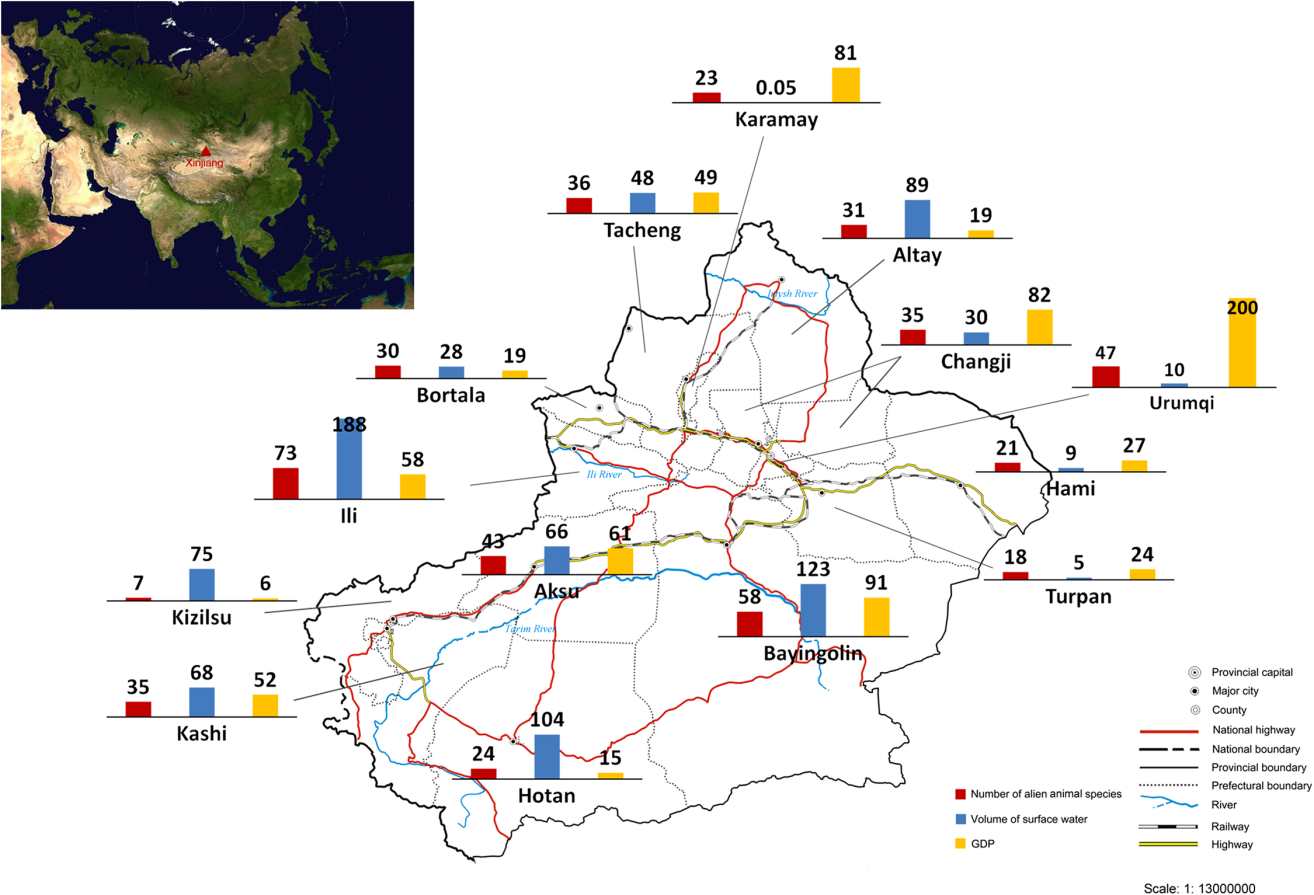
Number of recorded alien animal species, volume of surface water (10^7^ tons) and GDP (10^9^ Yuan RMB, equal to 1.61 × 10^8^ USD) of each prefecture of Xinjiang. The sketch map of Xinjiang is drafted by the Xinjiang Bureau of Surveying and Mapping, and was downloaded from the public service website of the Xinjiang Bureau of Surveying, Mapping and Geoinformation at: http://www.xjch.gov.cn/article/bzdt/index.shtml (accessed on December, 2014).

We calculated the number of recorded alien animal species in each of all 15 prefectures in Xinjiang (Fig. 2). The highest number of alien animal species was recorded in Ili Prefecture (73 species) in the western Tianshan Mountain area, followed by Bayingolin (58 species) which covers almost half of the Tarim Basin, and Urumqi (47 species) in the eastern Tianshan Mountain area. By contrast, fewer alien animal species occurred in the more arid Turpan and Hami prefectures (18 and 21 species, respectively) in the Turpan Basin. Only 7 species was recorded in Kizilsu Prefecture on eastern Pamir Plateau. Records of occurrence location were available for 119 of 128 alien animal species in our list. We used linear regression modelling to explain current distribution of alien animals in Xinjiang. The final model (Adjusted *R*^2^ = 0.929, DW = 2.163, *F* = 57.779, *P* < 0.0001, model summary in Table S1) included three variables: volume of surface water resources (β = 0.78, t = 9.706, partial correlation coefficient = 0.951, *P* < 0.0001), GDP (β = 0.729, t = 9.812, partial correlation coefficient = 0.952, *P* < 0.0001), and the share of transportation output in GDP (β = 0.237, t = 2.964, partial correlation coefficient = 0.684, *P* < 0.05). Potential variables with poor predictive performance were excluded during the modelling process. No collinearity existed among the variables in the final model (see Table S2).

### Temporal trends of aliens

**Figure 3:**
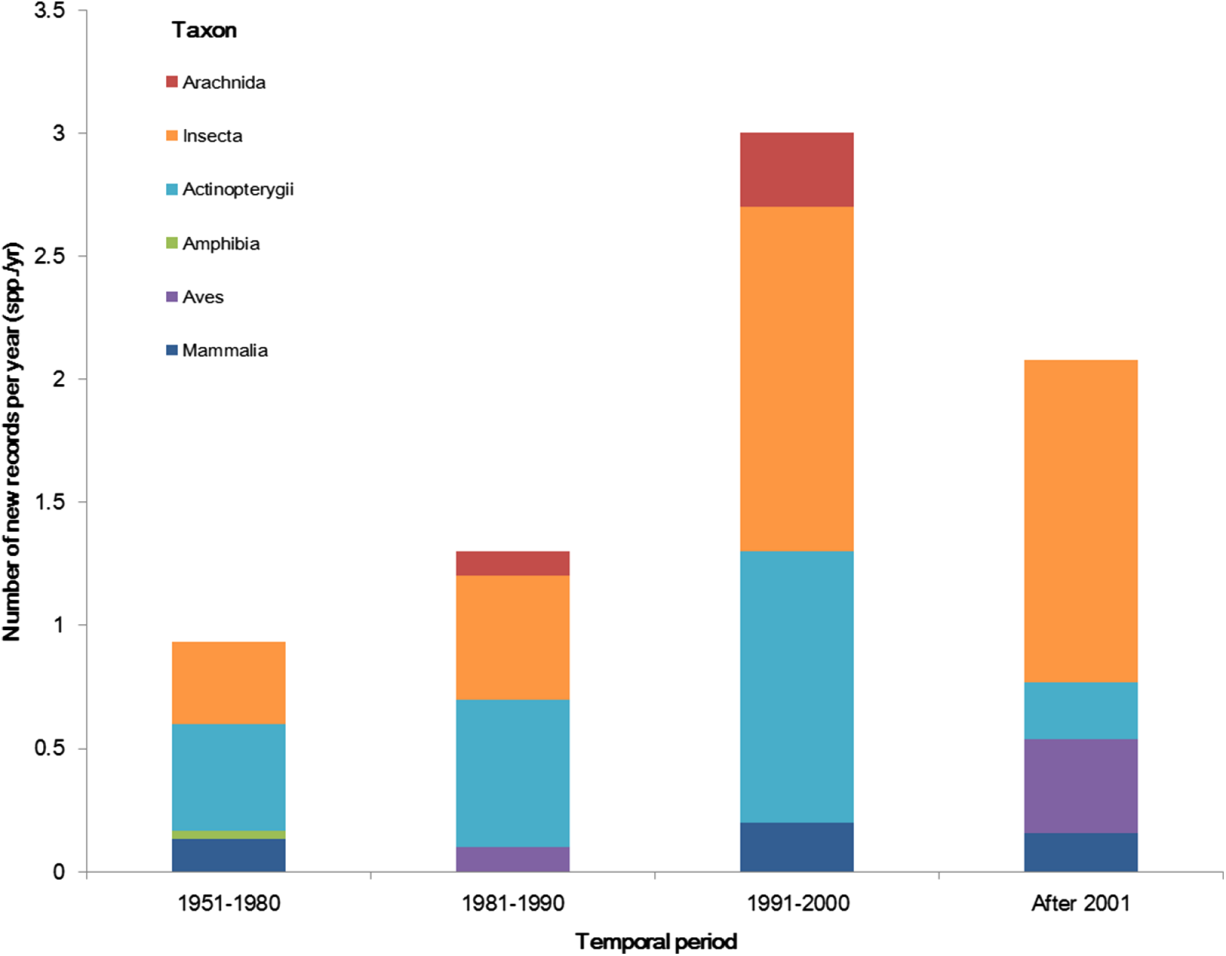
The temporal trend of alien animal species in the past 64 years. We considered the first recorded time of each species to graph the column figure. Species without such data were excluded.

**Figure 4:**
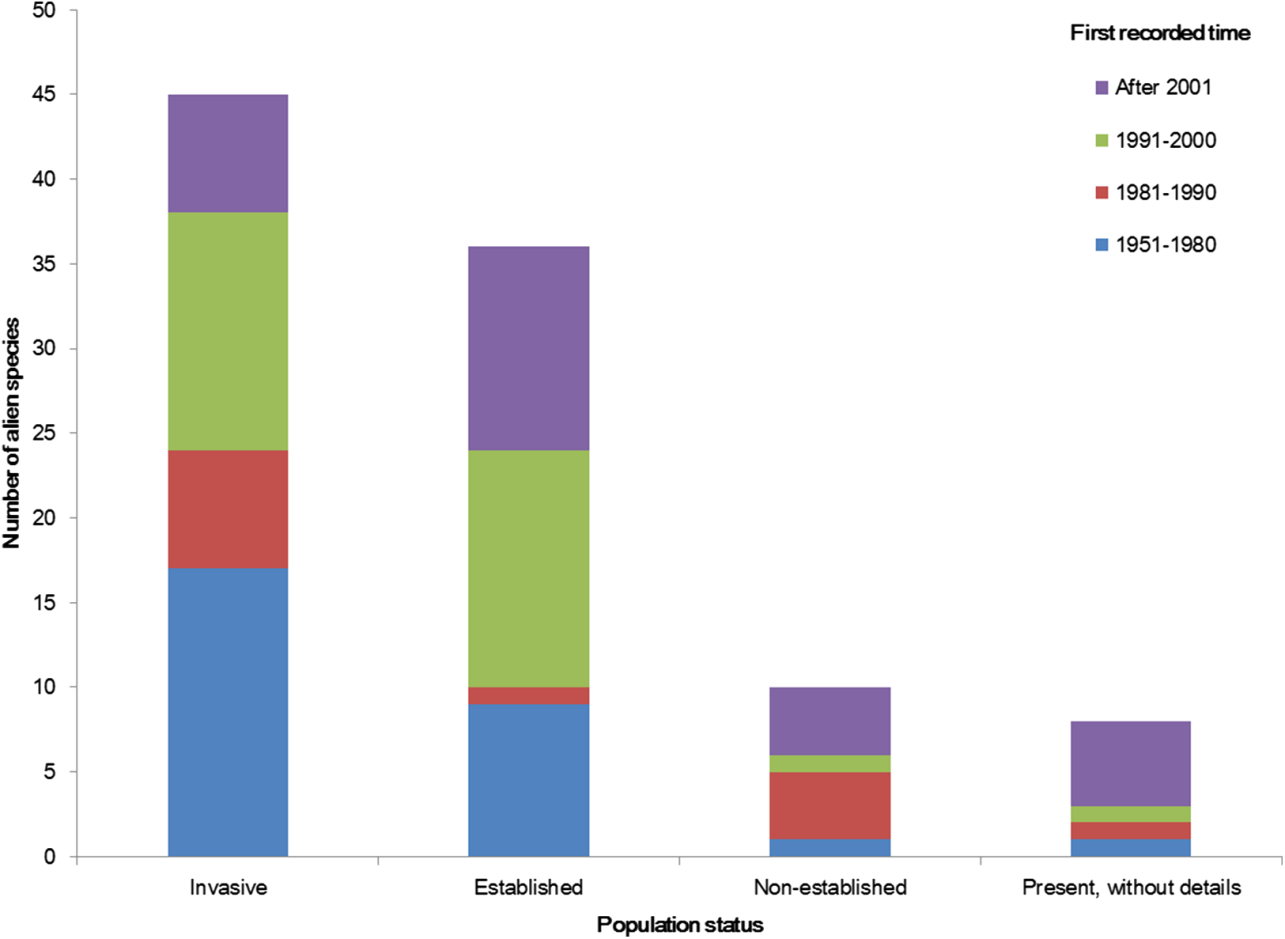
Population status of alien animal species detected in different temporal periods of the past 64 years. The first recorded time of each species was considered to graph the column figure. Species without such data were excluded.

The temporal trend of alien animal species in Xinjiang was illustrated in Fig. 3. From 1951 to 2000, the number of new records per year showed a gradual increase. The highest number of new alien species was recorded from 1991 to 2000, which was mainly attributed to growth of introduced fishes and arthropods (11 and 14 species, respectively). After 2001, introduction of alien animal species in Xinjiang slowed down slightly: new records of alien species dropped from 3 species per year during 1990s to ∼2.5 species per year from 2001 to 2014, most of which were insects. Almost half of alien animals introduced from 1991 to 2000 have become “invasive,” accounting for 44% of the total invasive animal species in the past 64 years. Comparatively, species introduced after 2001 showed less proportion of both “established” and “invasive” (Fig. 4). Temporal correlation was established between the share of transportation output in GDP (*r* = 0.886, *P* < 0.05), the share of imports in GDP (*r* = 0.821, *P* < 0.05) and number of new records of alien animal species per year from 1981 to 2010 (Table S3).

## Discussion

### Economy, environment and invasion process in Xinjiang

Human plays a key role in biological invasions at both regional and global scales (Weber, Sun & Li, 2008; McGeoch et al., 2010; Pyšek et al., 2010). Among the human activities, the ones involving economic development and trade, often diversify introducing pathways (Hulme et al., 2008), increase propagule pressure and bring disturbance to endemic biota (D’antonio et al., 1999; Lockwood, Cassey & Blackburn, 2005), and consequently boost the scale of alien species invasions to a significant extent. This paradigm was proved to be the case of Xinjiang. Using GDP as the surrogate of economic development status, we found that the number of alien animal species of each prefecture was largely explained by GDP rather than the total area. Features of the recipient environment also act on alien species invasion through influencing the survival and reproduction phases in alien species’ life history (Simberloff, 2009). For alien animal species arrived in arid Xinjiang, water resource is the other contributing factor of their distribution pattern. Many alien animals were recorded at locations situated near water sources, such as oases, rivers and cannels. Oases can serve as both harbours and dispersal corridors of alien animals. Up to now, most of extant oases in Xinjiang have been transformed to artificially irrigated mono-crop plantations. Such simple structured agroforestry biome provided empty niches for opportunistic alien species. Invasion of the silverleaf whitefly (*Bemisia tabaci*), which was detected in Xinjiang for the first time in 1998, was closely related to oasis agriculture mentioned above. Hitchhiking transported plantlets, pioneering populations of silverleaf whitefly settled in vegetable greenhouses in Urumqi and Turpan cities. They used greenhouses as refuges in cold winters, and spread to neighboring farmlands when greenhouse covers were removed in the following spring. Stepping on the connected agriculture oases and facilitated by commodities circulation, it expanded the distribution range to southern Tarim Basin in 2011 (Guo et al., 2012a, Fig. 5A). Besides oases, rivers, cannels and inland lakes facilitated invasion of alien fishes and riverain species (such as muskrat). Early in the 20^th^ century, many fish species of economic value were introduced into Balkhash Lake in Kazakhstan from the other Asian water systems such as the Aral Sea and Caspian Sea. Eight of these species (e.g. *Sander lucioperca*, *Leuciscus baicalensis*, and *Abramis brama orientlis,* see in Table 1) spread along the Ili River and drove endemic *Racoma argentatus* and *R. pseudaksaiensis* to extinction in the early 1990s (Ren, 1998). Transportation also contributed to the distribution of alien animal species in Xinjiang. However, its influence was much less significant than GDP and water resources, since areas with developed transportation may be lacking of enough suitable habitats, such as extremely arid prefectures like Turpan and Hami where we only found 18 and 21 alien species.

**Figure 5:**
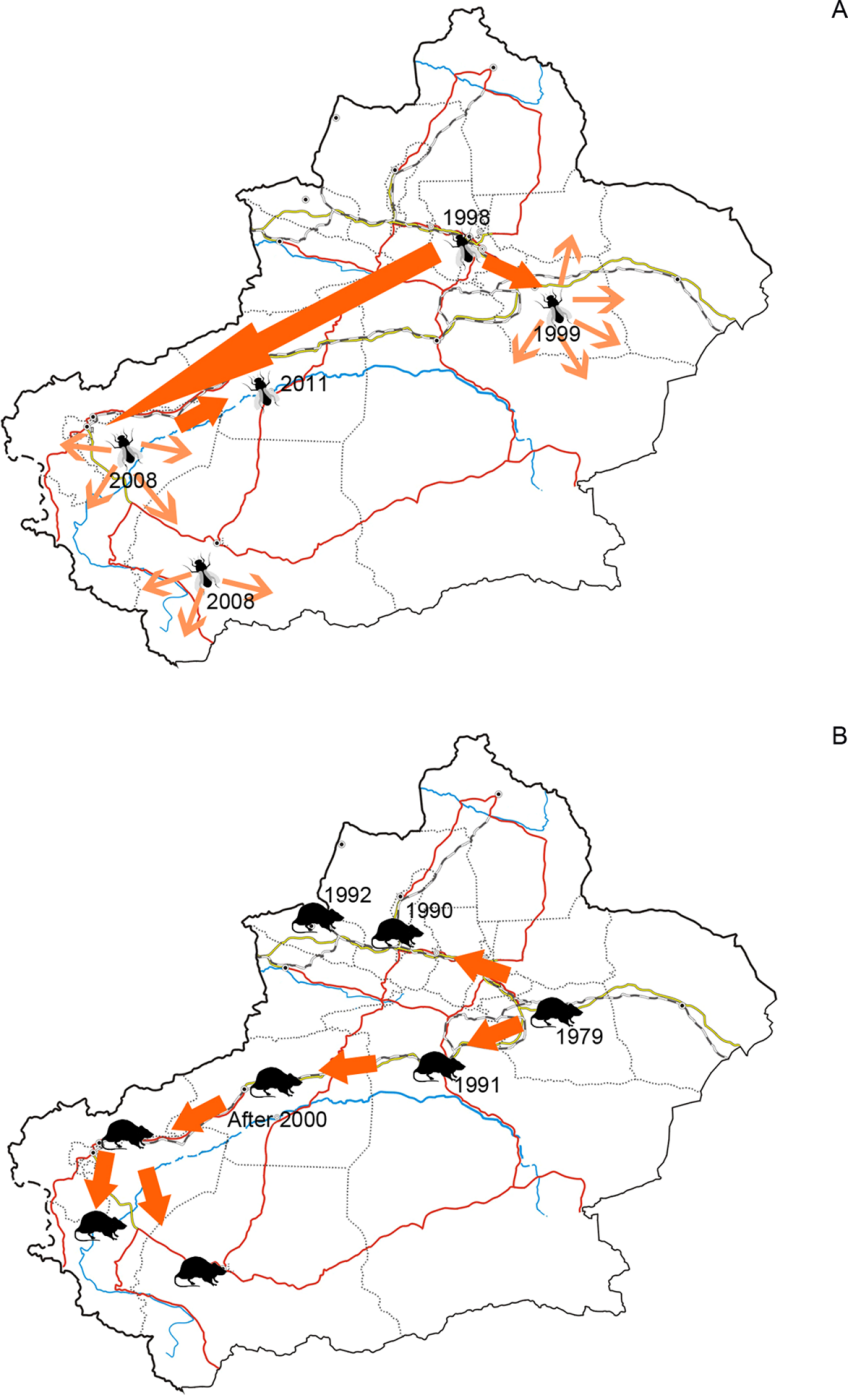
(A) Dispersal of silverleaf whitefly (*Bemisia tabaci*) in Xinjiang and (B) Dispersal of brown rat (*Rattus norvegicus*) in Xinjiang. Figure 5A was plotted according to Guo et al. (2012a) and 5B was plotted according to Zhang, Tao & Mahmut (1997).

The facts above indicated that distribution of alien animal species in the arid Xinjiang were jointly influenced by the level of economic development and the environmental suitability, which could be demonstrated by top-three hotspots of alien animals. Ili Prefecture, the first-rank hotspot of alien animal species, is a large oasis supplied with ample water resources from the Ili River. This area is characterized by flourishing economic activities such as stockbreeding, fruit industry, aquaculture and agriculture, which indicates a relative high propagule pressure; favourable natural conditions (e.g. humid and warm climate, lush forest and grassland) also increases the probability of successful establishment and reproduction of aliens. Our study suggested that Bayingolin Prefecture held the second highest number of alien animal species. By contrast to Ili, Bayingolin is largely covered by desert. Most of alien species in Bayingolin were recorded in the only oasis Kolra and the Bosten Lake. Kolra is a highly developed agricultural area with functions of important economic centre and transportation hub of southern Xinjiang; Bosten Lake is the largest aquaculture farm where 25 species of invasive fish, the highest among the water bodies in Xinjiang, were recorded (Lai, 2009; Peng et al., 2009). These facts thus jointly resulted in a great number of alien species in Bayingolin. Urumqi was found to be another hotspot of biological invasion with the third highest number of alien animals (47 species). This may be largely attributed to the fact that Urumqi is the most economically developed area in Xinjiang, and functions as the largest port of entry in the region with huge volume of foreign trade and highly developed transportation which are well-known drivers of alien species introductions (Hulme, 2007).

### The role of transportation and trade in the invasion process

The first record of alien animal species in Xinjiang dates back to 1900s, which was the *Apis mellifera* introduced for local honeybee industry (He et al., 1999). In the 1950s, human mediated introductions of alien animal species started to increase with flourishing domestication of animals of economic values. The obvious growth of alien animals appeared after 1980 (Fig. 3) along with the increasing traffic between Xinjiang and the world, owing to industrial and economic development facilitated by the “Reform and Opening-up Policy” implemented by Chinese government in 1978. For example, introduction of alien fishes in Xinjiang began with the national-wide propagating of Asian carps in early 1960s, which was of small scale because of limited transportation conditions and breeding facilities. However, an apprearent increase was observed in both the number of species and number of individuals released into main waterbodies of Xinjiang after late 1980s (Guo, Zhang & Cai, 2012b). Decades of introductions homogenized most of aboriginal fish fauna to a mixed European-Asian one (Wang, 1995; Ren, 1998; Ma et al., 2009). The Tarim is a closed inland river where fish fauna was historically composed of central Asian alpine species. For the development of local fishery, several alien fishes of economic values were repeatedly introduced to the Tarim River from east and south China in the latest half of 20^th^ century. Goldfishes (*Carassius auratus*), Wuchang breams (*Megalobrama amblycephala*) and Asian carps, outcompeted endemic species in resource utilization, and became dominant species in local fish communities. Fish invasion in Tarim River led to serious decline in populations of endemic fish, such as *Aspiorhynchus laticeps* and *Schizothor axbiddulphi* (Wang, 1995; Ma et al., 2009).

**Figure 6:**
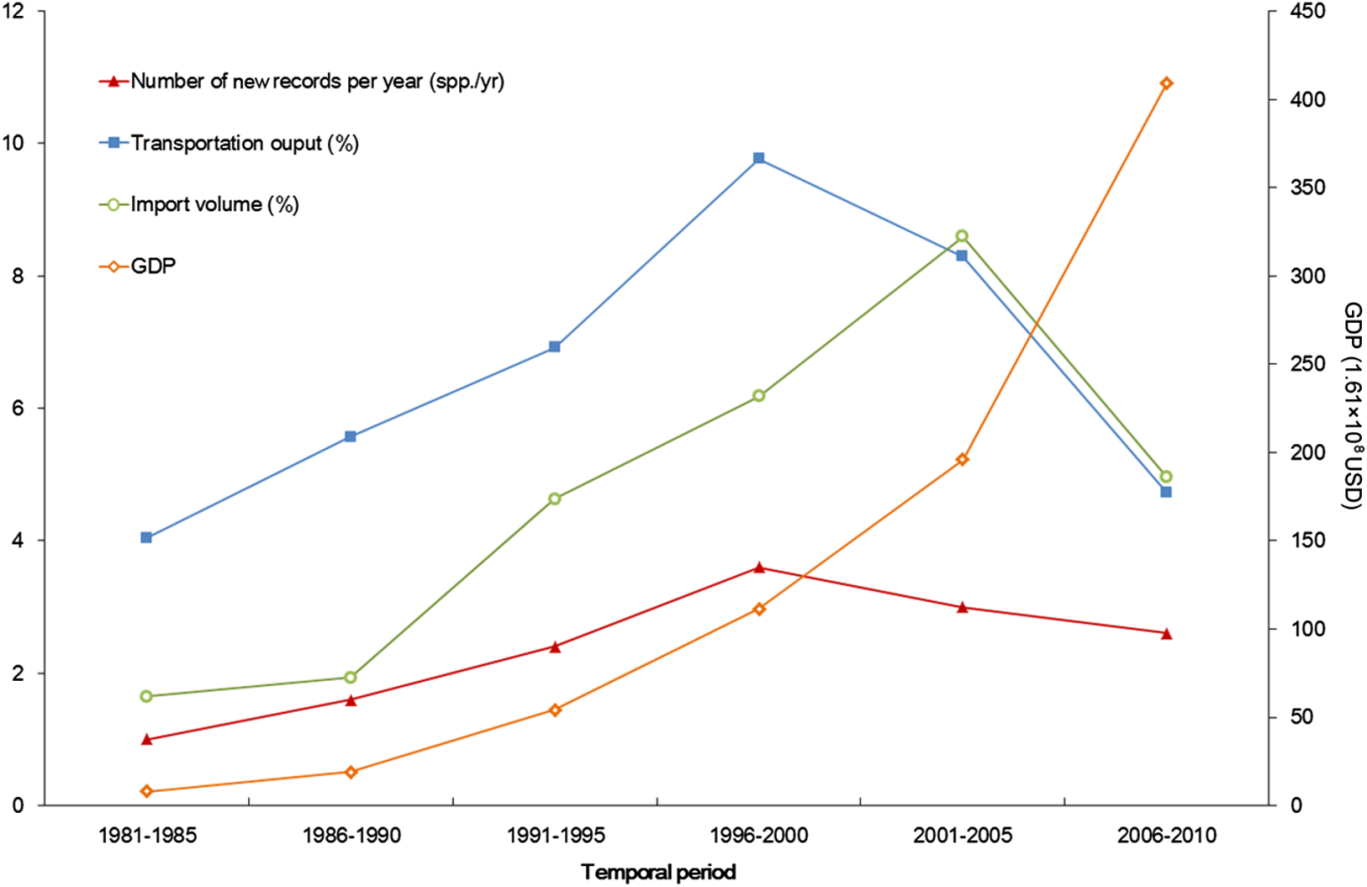
Trends of alien animal introduction and GDP, transportation development, and foreign trade. The x-axis begins with 1981 as systematic statistics in Xinjiang did not start until 1978.

Developed transportation is directly associated with high volume of domestic and international trade, which is considered to be a key indicator for aliens entry (EPPO, 2007). During the 1990s, transportation of Xinjiang experienced a remarkable boom illustrated in Fig. 6. Assisted by improved transportation conditions, a large number of plantlets were repeatedly introduced into Xinjiang for expanding local fruit industry (Bi, Liao & Li, 2002; Bi, Liao & Li, 2009). Together with the plantlets, alien arthropods hitchhiked to Xinjiang, which accounted for nearly 60% of the total alien animals recorded during the period (Fig. 3). In addition, along the extending transport networks, alien animals may spread widely resulting from transit of goods (Hulme et al. 2008). Successful establishment of the brown rat (*Rattus norvegicus*) was a typical case of mammal invasion facilitated by transportation development. The brown rat was first detected on a train from Beijing to Urumqi in 1975, and successively dispersed to the Western Tianshan Mountains, then to the Juggar Basin and the Tarim Basin along with extending railways in Xinjiang (Zhang, Tao & Mahmut, 1997). In the late 1990s, brown rats naturalized in almost the whole Xinjiang region (except Kizilsu), and growing age structure was observed in most of its populations (Zhang, Tao & Mahmut, 1997, Fig. 5B).

Besides transportation, increasing international trade from 1986 to 2005 (Fig. 6) is another factor correlated with temporal variation of alien animal introduction. In Xinjiang, 17 cross-border trade ports have been opened since 1973 (11 of them opened after 1990) along 5, 600km-long international boundary of the region. Opening air and land ports has boosted commodity exchange between Xinjiang and the other countries, which indicated the growth of likely vectors of alien species. The pace of transportation and trade development slightly slowed down in the latest decade. Meanwhile, severe ecological and economic impacts of invasive species (Guo et al., 2012a; Liu, Li & Zhang, 2012; Tang, Brown & Karjan, 2012) have stimulated a series of fortified prohibitive measures, such as veterinary quarantine and pest controls (Xue, Fan & Zhang, 2004). According to the reports from Xinjiang Entry-exit Inspection and Quarantine Bureau (2014), from 2000 to 2010, the number of alien animal species (or genera) intercepted in imported plants, merchandises and luggage increased from 22 to 50 species (or genera) per year, accumulating to over 68 species (or genera) in total. All above reined the fast pace of alien introduction but did not change the situation significantly. In addition, the latest status was more likely to be underestimated, because many of the newly arrived animals were at early stages of invasion process and therefore hard to detect within a relatively short time span.

### Gaps in knowledge

**Figure 7:**
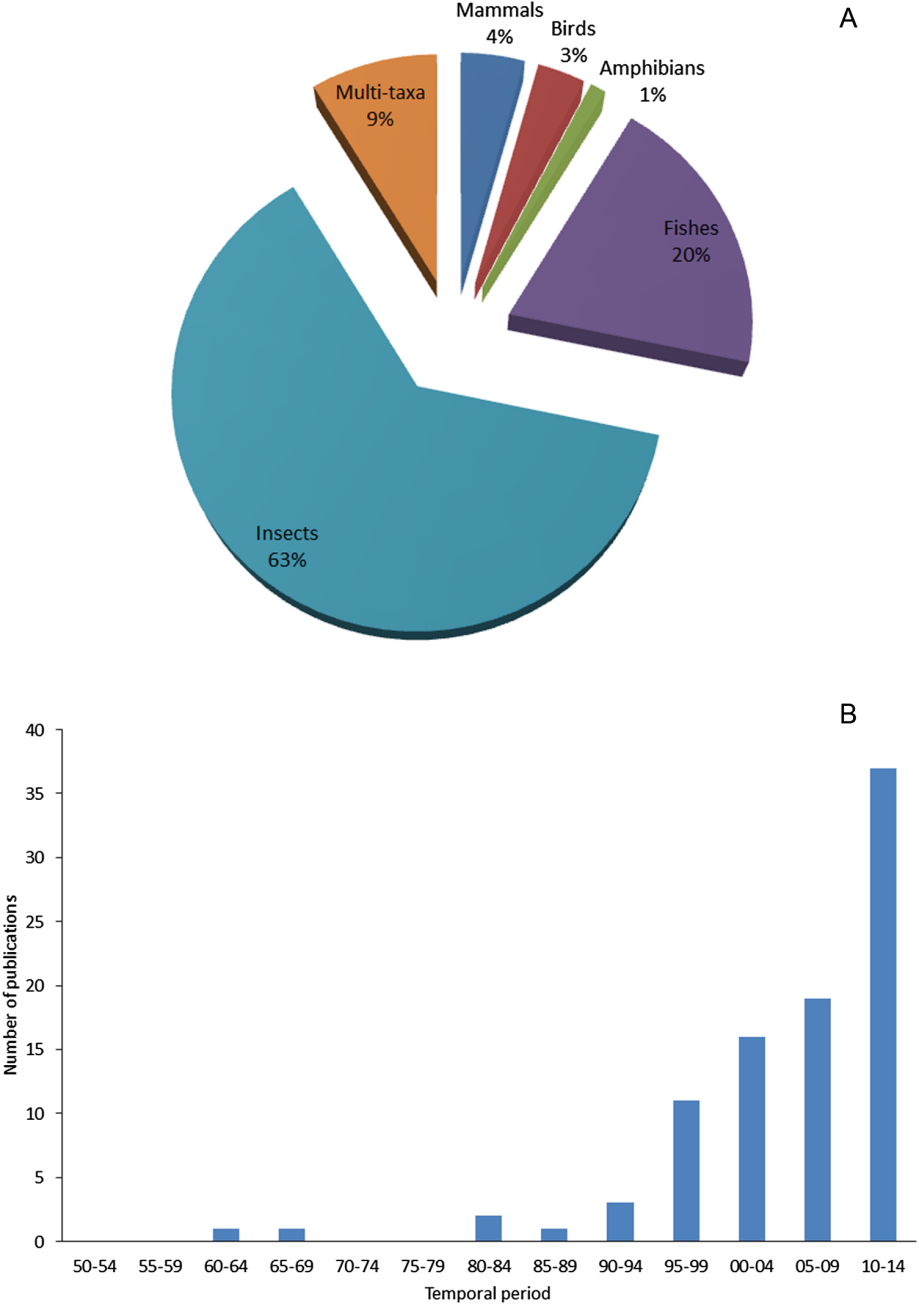
(A) Taxonomic structure of case studies on animal invasions in Xinjiang and (B) Trend of the number of publications on animal invasions in Xinjiang during 1950 to 2014.

By comparing the number of literatures for data extracting among taxa, we found an evident majority of specific studies on alien insects. In contrast, less studies on alien fishes, much less on alien mammals and birds were found: for 91 selected literatures, 63% focused on insects, 20% on fishes, only 4% on mammals and 3% on birds (Fig. 7A). Impact is the major factor of a species being chosen as a subject of study (Pyšek et al., 2008). As to Xinjiang, alien insects, especially pests of agriculture or fruit industry, are more likely to cause seriously economic loss in a relatively short period than other taxa. Consequently, the local government is willing to invest more effort to studies on such species for the sake of mitigating those economic impacts. Unequal financial support and resource distribution largely explained the bias in research intensity of different taxa. During the process of data mining, we were able to access an inventory of invasive arthropod pests in Xinjiang by retrieving peer-reviewed journals, and also gathered information on invasive insects covering aspects of invasion mechanisms, risk assessment, management and eradication from both English and Chinese literatures. However, limited knowledge was available on less influential (or harmful) taxa. Information on alien birds and reptiles were the scarcest; we did not find any specific reports on these taxa. As for alien mammals and fishes, we found case-specific studies focused on few species threatening agricultural production or human health, such as brown rats (Zhang, Tao & Mahmut, 1997) and pond smelts (*Hypomesus olidus*) (Lai, 2009). Besides taxonomic bias in research on recorded aliens, we found that research on this field in Xinjiang apparently lagged behind. 75% of the total 91 literatures were published after 2000, but only 17 papers related to alien animal species were published during 1990s when the number of new records of aliens reached the peak (Fig. 7B). It also showed that recent studies focused more on later stages of invasion process such as management and eradication rather than initial prevention stage (see Supplemental Reference), since scientific research on biological invasions in Xinjiang was strongly directed by the needs of reducing losses caused by alien species.

Through the process of information gathering and scrutiny, we found that documentation of alien animals in Xinjiang was still far from sufficient. Long-term monitoring on population dynamics and invasion pathways of alien animals is necessary for scientific management. Market investigations on local husbandry and pet trade (Su, Cassey & Blackburn, 2014) are feasible complementary measures of alien animal monitoring, especially for mammals and birds. Additionally, “event-level” information (e.g. propagule pressure) should be recorded for understanding early stages of the invasion process. We also recommended taxonomically balanced study of alien species in future. Enrichment of knowledge on alien species will help to develop sound policies on biological invasions. This is vital for a research-lagged region like Xinjiang, because less-informed policies might aggravate current unequal allocation of resource for scientific research. Insufficient resource and supports will hinder researchers from concealing risks of less-concerned species with potential invasiveness. Successful control of biological invasions requires co-production of knowledge within a co-management governance system (Moon, Blackman & Brewer, 2015). For a multi-ethnic inhabited region such as Xinjiang, community stakeholders should be viewed as equal partners in policy making and action implementing, in order to prevent dissent and conflict caused by different cultural backgrounds.

Recently, alien species have been testified to hold a greater likelihood of becoming “invasive” pertaining to a population level in many taxa (Blackburn et al., 2011; Simberloff et al., 2012; Paolucci, MacIsaac & Ricciardi, 2013; Hassan & Ricciardi, 2014). Therefore, potential risks of alien species deserve consideration in management decisions. In this context, to mitigate ecosystem impact and economic loss, priority should be transferred from eradication to prevention and early detection which hold visible and long-term effectiveness in invasive species management (Keller, Lodge & Finnoff, 2007). In cases such as the least-concerned alien herpeto-species, congener diversity of recipient localities is recommended to assist for evaluating risks and prevention, when a certain species is entrained on introductions or proposed for introduction (Liu et al., 2014). Considering the uncertain and delayed impacts of invasions (Simberloff et al., 2013; Essl et al., 2015), we suggested paying attention to alien animals that have yet not become “invasive” in Xinjiang but are included in Global Invasive Species Database (ISSG, 2014), such as the American mink (*Neovison vison*), racoon dog (*Nyctereutes procyonoides*), and snakehead fish (*Channa argus*), since their invasiveness has been witnessed by comprehensive and significant impacts in other regions of the world. Monitoring their population dynamics and impacts will help for surveillance and timely eradication. Furthermore, we called for applications of risk assessment tools, for example, horizon scanning (Roy et al., 2014) and adaptive modelling (Uden et al., 2015) based on ecological niche models (ENMs, Jiménez-Valverde et al., 2011), to predict the hazards posed by alien species, especially those with no prior invasion history in Xinjiang, as well as the vulnerability of native biodiversity to emerging disturbance. The tools will contribute to prioritization of risks brought by alien species for developing management strategies on animal invasions through systematically examining potential threats and opportunities in the future. Finally, the role of oasis playing in animal invasions in arid zone is significant, as it is often environmentally suitable and exposes to high propagule pressure. This fact needs special concern during the execution of the China’s “New Silk Road” economic belt strategy, because economic drivers will accelerate introductions of invasive alien species in emerging oasis economies which are stepping stones of development in arid zones.

## Conclusions

Xinjiang, located in the arid zone of Asia, is a poorly-known region when it comes to invasion ecology. This study confirmed that occurrence, establishment, spread of alien animal species were associations with human and environmental factors. During the past six decades, development of transportation and trade facilitated alien animals entering this closed inner-land region; local economic development level and availability of water resources contributed to distribution pattern of alien animals in Xinjiang. Oases were important battlefields for prevention and management of invasive animals in the arid environment. Based on research status of animal invasions in Xinjiang, we suggested reallocating more efforts to alien population monitoring, particularly for species with highly invasive potential; for those invasive species, impact and risk assessment were urgently needed.

## Supplemental Information

10.7717/peerj.1545/supp-1 Supplemental Information 1 Linear regression model summary on alien species distribution and potential variable. Dependent of each model is the number of alien species per prefecture. a: SW, volume of surface water resource; GDP, gross domestic products; TS, the share of transportation output in GDP. b: *R*, multiple correlation coefficient; *R^2^*, determination coefficient, reflects the proportion in variance of response variables is explained by predictors. As *R^2^* will rise with number of variables increasing in multiple regression analysis, we used adjusted *R^2^* for testing the fitness of the model. Durbin-Watson parameter (DW), reflects independence of residuals in the model. We consider estimations and conclusions based on a certain model are credible when DW approximates to 2.

10.7717/peerj.1545/supp-2 Supplemental Information 2 Coefficients and excluded variables in the linear regression model. **a:** B, unstandardized regression coefficients; β, standardized regression coefficients, used for comparing contribution of predictors to the response variable; t, used for testing predictive result of variables. An ideal predictor should hold t value above 2 or below −2. **b:** we used 15 as the threshold of condition index, of which value above 15 indicated a collineray between variables. Besides, collineary is considered existing if the tolerance and eigenvalue approximated to 0. **c:** indicated *P* < 0.0001 **d:** we excluded potential predictors from the final model using the standard of −2 < t < 2 and *P*> 0.05. Area of the prefecture, wetlands and used land, GDP, share of transportation output and imports in GDP were log transformed before analysis to normalize the data.

10.7717/peerj.1545/supp-3 Supplemental Information 3 Correlation Analysis on temporal trends of alien introductions and human factors. a: GDP, gross domestic products; TS, the share of transportation output in GDP; IS, the share of imports in GDP. * Correlation is significant at the 0.05 level (2-tailed).

10.7717/peerj.1545/supp-4 Supplemental Information 4 Raw data.

10.7717/peerj.1545/supp-5 Supplemental Information 5 Supplemental Reference List. We listed literature, reports and databases that were used for data extraction but were not referenced in the **Main Text** as part of the raw data.

## Additional Information and Declarations

### Data Deposition

The following information was supplied regarding data availability: Raw data can be found in the Supplemental Information.
