# Supplementary material for: Unveiling the status of alien animals in the arid zone of Asia
Source: PeerJ. 2016 Jan 12;4:e1545. doi: 10.7717/peerj.1545 (PMC4715455; doi:10.7717/peerj.1545)
Supplement: Supplemental Information 1 — Dependent of each model is the number of alien species per prefecture. a: SW, volume of surface water resource; GDP, gross domestic products; TS, the share of transportation output in GDP. b: R, multiple correlation coefficient; R2, determination coefficient, reflects the proportion in variance of response variables is explained by predictors. As R2 will rise with number of variables increasing in multiple regression analysis, we used adjusted R2 for testing the fitness of the model. Durbin-Watson parameter (DW), reflects independence of residuals in the model. We consider estimations and conclusions based on a certain model are credible when DW approximates to 2. [file peerj-04-1545-s001.docx]

|  | **SW ^a^** | **GDP ^a^** | **TS ^a^** | ***R* ^b^** | ***R*^2 b^** | **Adjusted *R^2^* ^b^** | ***F*** | ***P*** | **Durbin-Watson ^b^** |
| --- | --- | --- | --- | --- | --- | --- | --- | --- | --- |
| **Model 1** |  | ● |  | 0.654 | 0.428 | 0.380 | 8.983 | 0.011 | 2.597 |
| **Model 2** | ● | ● |  | 0.947 | 0.898 | 0.879 | 48.170 | ＜0.0001 | 2.090 |
| **Model 3** | ● | ● | ● | 0.972 | 0.945 | 0.929 | 57.799 | ＜0.0001 | 2.163 |
