# Supplementary material for: Unveiling the status of alien animals in the arid zone of Asia
Source: PeerJ. 2016 Jan 12;4:e1545. doi: 10.7717/peerj.1545 (PMC4715455; doi:10.7717/peerj.1545)
Supplement: Supplemental Information 2 — a: B, unstandardized regression coefficients; β, standardized regression coefficients, used for comparing contribution of predictors to the response variable; t, used for testing predictive result of variables. An ideal predictor should hold t value above 2 or below −2. b: we used 15 as the threshold of condition index, of which value above 15 indicated a collineray between variables. Besides, collineary is considered existing if the tolerance and eigenvalue approximated to 0. c: indicated P < 0.0001 d: we excluded potential predictors from the final model using the standard of −2 < t < 2 and P> 0.05. Area of the prefecture, wetlands and used land, GDP, share of transportation output and imports in GDP were log transformed before analysis to normalize the data. [file peerj-04-1545-s002.docx]

| **Model** | | | **B** ^a^ | β^a^ | **T** ^a^ | ***P*** | **Correlations**  **(partial)** | **Collinearity Diagnostics** ^b^ | | |
| --- | --- | --- | --- | --- | --- | --- | --- | --- | --- | --- |
|  |  |  |  |  |  |  |  | **Tolerance** | **Eigenvalue** | **Condition Index** |
| **1** | **(Constant)** | | -150.898 |  | -2.437 | 0.031 |  |  |  |  |
|  | **GDP** | | 12.198 | 0.654 | 2.997 | 0.011 | 0.654 | 1.000 | 1.998 | 1.000 |
|  |  | |  |  |  |  |  |  |  |  |
| **2** | **(Constant)** | | -184.289 |  | -6.635 | 0.000^c^ |  |  |  |  |
|  | **GDP** | | 13.530 | 0.726 | 7.479 | 0.000^c^ | 0.914 | 0.989 | 2.680 | 1.000 |
|  | **SW** | | .219 | 0.689 | 7.098 | 0.000^c^ | 0.906 | 0.989 | .318 | 2.902 |
|  |  | |  |  |  |  |  |  |  |  |
| **3** | **(Constant)** | | -197.120 |  | -9.089 | 0.000^c^ |  |  |  |  |
|  | **GDP** | | 13.585 | 0.729 | 9.812 | 0.000^c^ | 0.952 | 0.989 | 3.518 | 1.000 |
|  | **SW** | | 0.248 | 0.780 | 9.706 | 0.000^c^ | 0.951 | 0.845 | 0.418 | 2.902 |
|  | **TS** | | 7.150 | 0.237 | 2.964 | 0.014 | 0.684 | 0.853 | 0.063 | 7.491 |
|  | | | | | | | | | | |
| **Excluded Variables^d^** | | **A** |  |  | -0.452 | 0.662 | -0.149 | 0.702 |  |  |
|  |  | **WL** |  |  | 1.363 | 0.206 | 0.414 | 0.494 |  |  |
|  |  | **T** |  |  | -1.075 | 0.310 | -0.337 | 0.921 |  |  |
|  |  | **P** |  |  | 0.165 | 0.873 | 0.055 | 0.836 |  |  |
|  |  | **RT** |  |  | 1.268 | 0.236 | 0.389 | 0.608 |  |  |
|  |  | **LU** |  |  | 0.303 | 0.769 | 0.100 | 0.645 |  |  |
|  |  | **IS** |  |  | 1.413 | 0.191 | 0.426 | 0.765 |  |  |
