# Supplementary material for: Unveiling the status of alien animals in the arid zone of Asia
Source: PeerJ. 2016 Jan 12;4:e1545. doi: 10.7717/peerj.1545 (PMC4715455; doi:10.7717/peerj.1545)
Supplement: Supplemental Information 3 — a: GDP, gross domestic products; TS, the share of transportation output in GDP; IS, the share of imports in GDP. * Correlation is significant at the 0.05 level (2-tailed). [file peerj-04-1545-s003.docx]

|  |  | **GDP^a^** | **TS^a^** | **IS^a^** |
| --- | --- | --- | --- | --- |
| Annual rate of new alien species | Pearson Correlation (*r*) | 0.747 | 0.886* | 0.821* |
|  | *P* | 0.088 | 0.019 | 0.045 |
