# Supplementary material for: Unveiling the status of alien animals in the arid zone of Asia
Source: PeerJ. 2016 Jan 12;4:e1545. doi: 10.7717/peerj.1545 (PMC4715455; doi:10.7717/peerj.1545)
Supplement: Supplemental Information 5 — We listed literature, reports and databases that were used for data extraction but were not referenced in the Main Text as part of the raw data. [file peerj-04-1545-s005.docx]

**Supplemental Reference**

We listed literature, reports and databases that were used for data extraction but were not referenced in the **Main Text** as Supplemental Reference.

Ahtam U, Liu J, Aniwar K, Tursunjiang Y, Liu W, Rao P, Shen Y. 2011. Preliminary survey on growth and decline of natural enemies and pest insects in Hetian Region of Xinjiang, China. *Xinjiang Agricultural Sciences*, **48**, 252-256. (in Chinese)

Barhatguri. 2009. Impacts and management of Cydia pomonella in Xinjiang. *Protection Forest Science and Technology*, **4**, 58. (in Chinese)

Cai L, Li H, Zhang R, Adakbek, Liu J. 2008. The preliminary inverstigation of aquatic organisms in Huoerguosi River. *Journal of Hydroecology*, **6**, 39-43. (in Chinese)

Chen W, Yu J, Xu Y, Liu X. 2006. First occurrence of Eriosoma lanigerum in Xinjiang. *Xinjiang Agricultural Sciences*, **43**, 309-309. (in Chinese)

CMIAS, IPP Database of Invasive Alien Species of China (in Chinese). In: Centre of Management of Invasive Alien Species, Ministry of Agriculture of People Republic China and Institute of Plant Protection, Chinese Academy of Agriculture Science. Avilable at http://www.chinaias.cn/wjPart/index.aspx (accessed 14 October, 2014)

Du B. 2000. Research on distribution area and suitable conditions of Diuraphis noxia mordv. in Xinjiang. *Xinjiang Agricultural Sciences*, 82-85. (in Chinese)

Gozlan RE, Andreou D, Asaeda T, Beyer K, Bouhadad R, Burnard D, Caiola N, Cakic P, Djikanovic V, Esmaeili HR, Falka I, Golicher D, Harka A, Jeney G, Kováč V, Musil J, Nocita A, Povz M, Poulet N, Virbickas T, Wolter C, Serhan Tarkan A, Tricarico E, Trichkova T, Verreycken H, Witkowski A, Guang Z C, Zweimueller I, Robert BJ. 2010. Pan-continental invasion of Pseudorasbora parva: towards a better understanding of freshwater fish invasions. *Fish and Fisheries*, **11**, 315-340.

Guo W, Yang Z. 1963. Cultivation of minks. *Xinjiang Agricultural Sciences*, **12**, 004. (in Chinese)

Guo W, Ma Q. 2004. The situation and strategies of plant protection of Xinjiang in twenty-first century. *Xinjiang Agricultural Sciences*, **41**, 257-262. (in Chinese)

Guo W, Tuerxun, Xu J, Liu J, He J, Li J, Ma D, Wang J. 2010. Research on the identification of Colorado Potato Beetle and its distribution, dispersal and damage in Xinjiang. *Xinjiang Agricultural Sciences*, **47**, 906-909. (in Chinese)

Guo Y. 2002. Status of fishery development in Xinjiang, China. *Fishery Modernization*, 95. (in Chinese)

Guo Y, Zhang R, Li H. 2003. Degeneration causes of aboriginal fishes and measures for protecting them in Irtysh River, Xinjiang, China. *Arid Zone Research*, **20**, 152-154. (in Chinese, with English abstract)

He S, Wen J, Satar A, Tian C. 2010a. Research progress of quarantine pest *Carpomya vesuviana*. *Scientia Silvae Sinicae*, **46**, 147-154. (in Chinese)

He S, Zhu T, Satar A, Yu F, Wen J, Tian C. 2009. Occurrence of *Carpomya vesuviana* in Turpan region. *Chinese Bulletin of Entomology*, 930-934. (in Chinese)

He S, Zhu Y, Satar A, Wen J, Chen M, Tian C. 2010b. Pest risk assessment of Carpomya veusuviana in China. *Scientia Silvae Sinicae*, **47**, 107-116. (in Chinese)

He X, Lv G, Qin L, Maksat A, Liu H, Zhang, X. 2013. A comparison of species diversity in Turkmenistan and Xinjiang. *Arid Land Geography*, **36**, 623-630. (in Chinese)

Hu J, Angeli S, Schuetz S, Luo Y, Hajek AE. 2009. Ecology and management of exotic and endemic Asian longhorned beetle Anoplophora glabripennis. *Agricultural and forest entomology*, **11**, 359-375.

Hu L, Tian C, Zhu Y, Zhou Z, Ren L, Qi C. 2013. Biological characteristics of the ber fruit fly, *Carpomya vesuviana* (Diptera: Tephritidae). *Acta Entomologica Sinica*, **56**, 69-78.

Hu J, Wu W. 1989. *Spiders from agricultural areas of Xinjiang Uygur Autonomous Region, China*. Jinan: Shandong University Press. (in Chinese)

Huang D. 2002. Species, distributions and conservation of Acipenseriformes. *Tsinghua Science and Technology*, **7**, 416-420.

Huang W, Zhang C, Ren D. 2003 Test on control of *Parlatoria oleae* (Colvee) in field. *Xinjiang Agricultural Sciences*, **41**, 355-356. (in Chinese)

Huo T, Jiang Z, Karjan A, Ma B, Zhang L, Tang F, Cai L, Liu L. 2010. Status of fishes distribution and species diversity in Irtysh River Basin in China. *Journal of Hydroecology*. (in Chinese)

IPP China Agriculture Pests Information System (in Chinese). In. Institute of Plant Protection, Chinses Academy of Agriculture Sciences. Avilable at http://www.agripests.cn/ (accessed 25 March, 2015)

Ji Y, Ji R, Huang R. 2004. Invasive species - Agrilus Mali Matsumura and damage in Xinjiang. *Xinjiang Agricultural Sciences*, **41**, 31-33. (in Chinese)

Jiang S, Guo T, Ren D, Zhou L, Samat. 2003. Occurrence and management of *Cacopsylla chinensis*. *China Fruits*, 7-10. (in Chinese)

Jiang W, Cao W, Feng Y, Fang J, Li Y. 2010. Inter-regional biological invasion in China: present status and countermeasures. *Chinese Journal of Ecology*, **29**, 1451-1457. (in Chinese, with English abstract)

Jiang W, Lu W, Guo W, Xia Z, Fu W, Li G. 2012. Chlorantraniliprole susceptibility in Leptinotarsa decemlineata in the north Xinjiang Uygur autonomous region in China. *Journal of economic entomology*, **105**, 549-554.

Jiao S, Yue C, Zhang X, Liu A, Arimu. 2009. Record and narrate on exotic harmful species of forest in Xinjiang. *Xinjiang Agricultural Sciences*, **46**, 95-101. (in Chinese)

Li B, Li Y. 2000. First spread of Janus piri in Kashgar, Xinjiang. *Northern Horticulture*, **2**, 005. (in Chinese)

Li C. 2013. Research on Diffusion Rule of *Leptinotarsa decemlineata* Based on GIS in Xinjiang, China. PhD Thesis. Chongqing, China: Southwest University.

Li C, Cheng D, Liu H, Zhang Y, Sun J. 2013. Effects of temperature on the distribution of the Colorado Potato Beetle (*Leptinotarsa decemlineata*)—— effect of high temperature on its emergence in Turpan, Xinjiang. *Scientia Agricultura Sinica*, **46**, 737-744.

Li C, Zhang Z, Guo W, Zhang Y, Sun J, Cheng D, Liu H. 2011. The relationship between the occurrence of Colorado Potato Beetle, *Leptinotarsa decemlineata*, and rivers based on GIS: a case study of Shawan Country, Xinjiang. *Acta Ecologica Sinica*, **31**, 6488-6494.

Li H, Zhang R, Cai L, Guo Y. 1998. Development and protection of economics fish in Yili River. *Arid Zone Research*, **16**, 14-18. (in Chinese, with English abstract)

Li S, Dai D, Zhang S, Ma G, He Z, Gao S. 1966. Notes on a collection of fishes from north Xinjiang, China. *Acta Zoologica Sinica*, **18**, 41-56.

Li S. 1981. *Distribution and zoogeographic division of freshwater fish in China*. Beijing: Science Press. (in Chinese)

Li W, Liao L, Xie Y, Yang B, Li J. 1994. Study on the present of migration rodent─Brown Rat (*Rattus norvegicus*) in Xinjiang. *Chinese Journal of Vector Biology and Control*, **5**, 31-33. (in Chinese)

Liang H, Zhang R, Yan P, Wen Y. 1999. Distribution of the Russian Wheat Aphid and its natural enemies at different elevations. *Acta Entomologica Sinica*, **42**, 78-85.

Liang L, Yu H, Liu X, Zhang J, Chen N, Yang D. 2010. Analysis of suitability of the codling moth, *Cydia pomonella* in China. *Plant Protection*. (in Chinese)

Liu C, Zhao L, Ni Y. 2013. A Study on the Biological Characteristics of Alfalfa Seed Chalcid, *Bruchophagus roddi* (Gussakovsky). *Journal of Xinjiang Agricultural University*, **36**, 234-240. (in Chinese)

Liu N, Li Y, Zhang R. 2012. Invasion of Colorado potato beetle, *Leptinotarsa decemlineata*, in China: dispersal, occurrence, and economic impact. *Entomologia Experimentalis et Applicata*, **143**, 207-217.

Luan F, Zheng W, Li F, Ma D. 2006. Occurrence and spatial distribution of *Erythroneura apicalis* (Nawa), (Homoptera: Cicadellidae) populations in Turpan area. *Acta Entomologica Sinica*, **49**, 416-420.

Ma M. 2010. Bird expansion to east and the variation of geography distribution in Xinjiang, China: cases of the invasive species as greenfinch and myna. *Arid Land Geography*, **33**, 540-546. (in Chinese, with English abstract)

Ma N, Wang H, Zhang W, Simayi Y, Luo L, Ma D. 2008. The damage dynamics and spatio-temporal distribution of *Bemisia tabaci* (Gennadius) in cotton field based on GIS analysis in Xinjiang. *Acta Ecologica Sinica*, **28**, 2654-2662.

Ma Q. 2009. Occurring and controlling of *Aphis gossypii* in cotton filds in Xinjiang. *Journal of Xinjiang Agricultural University*, 47-50. (in Chinese)

Ma Y, Guo Y, Chen P, Xie C, Qi F, Niu J. 2013. Fauna composition and distribution of fish in the Kaidu River of Xinjiang Uygur Autonmous Region. *Freshwater Fisheries*, **43**, 21-26. (in Chinese)

MacKinnon J, Philipps K, He F. 2001. *A field guide to the birds of China*. Oxford: Oxford University Press.

Pan H, Ye R. 1992. Population of *Ondatia zibethica* in Xinjiang and utilizing. *Xinjiang Agricultural Sciences*, **3**, 017. (in Chinese)

Peng Y, Xue D, Wu L, Xue F. 2009. Mechanism of biological invasions in Bosten Lake, Xinjiang. *Jiangsu Agricultural Sciences*, **5**, 300-303. (in Chinese)

Qin X, Ma D, Zhang Y, Li G, Wang P. 2006. Impacts of *Cydia pomonella* in Northwest China. *Plant Quarantine*, **20**, 95-96. (in Chinese)

Ren M. 1998. Fishes of River Ili. *Chinese Journal of Fisheries*, **1**, 001.

Ren M. 2002. Fishes and fish fauna composition in Irtysh River, China. *Arid Zone Research*, **19**, 62-66. (in Chinese, with English abstract)

Rong L. 1989. Reasons of *Aphis gossypii* Glover.'s boosting in Xinjiang. *Xinjiang Agricultural Sciences*, **6.** (in Chinese)

Song M, Zhou L. 1990. A new pear pest -first report of *Janus piri*. *Xinjiang Agricultural Sciences*, 261-262. (in Chinese)

Wang C, Zhang J, Yue C, Zhang X, Wang Y, Liu A, Mamat A. 2012a. Risk assessment of *Pseudaulacaspis pentagona* in Xinjiang. *Jiangsu Agricultural Sciences*, **40**, 117-119. (in Chinese)

Wang J, Mamat A, Chen Q, Zhang L, Li H, Zhao Y, Ma D. 2012b. Introduction and control of *Phenacoccus solenopsis* Tinsley in Xinjiang. *Plant Quarantine*, **4**, 031. (in Chinese)

Wang S, Li J, Li K, Wang P, Li G. 2010. A new record species of leafhoppers from wine grapes in Xinjiang, China. *Plant Protection*, **36**, 168-171. (in Chinese)

Wei Z, Zhao L, Yang S. 2010. Advances of Studies on *Pseudaulacaspis pentagona* (Targioni-Tozzetti). *Xinjiang Agricultural Sciences*, **2**, 026. (in Chinese)

Wu F, Guo Y. 1984. Distribution, lifecycle and economic values of Ectobiidae in China. *Acta Entomologica Sinica*, **27**, 439-443.

Wu W, Lv Z, Wang D, Zhang J, Yan S. 2006. Dynamics of *Aphis gossypii* and its predatory natural enemies in organic agricultural cotton filed. *Chinese Journal of Ecology*, **25**, 1173-1176. (in Chinese, with English abstract)

Wu Z, Qu W, Zhang Z, Zhao L, Li Z. 2012. The potential geographical distribution of *Tychius medicaginis* based on the CLIMEX in China. *Plant Protection*, **38**, 63-66. (in Chinese)

Xi Y. 1997a. Burst of *Eulecanium gigantea* and management in Xinjiang. *Plant Quarantine*, **11**, 340-341. (in Chinese)

Xi Y. 1997b. Introduction of *Eulecanium gigantea* and reasons of its burst in Xinjiang. *Forest Pest and Disease*, **4**, 47. (in Chinese)

Xi Y, Song Y. 1998, Distribution, host and impacts of *Eulecanium gigantea* in Xinjiang. *Forest Pest and Disease*, 18-20. (in Chinese)

Xi Y, Bai Y. 2000. Control index of *Eulecanium gigantea.* *Forest Pest and Disease*, **19**, 15-17. (in Chinese)

Xiao H, Kai W. 1980. Establishment of Grass Carp (*Ctenopharyngodon idellus*) and Sliver Carp (*Hypophthalmichthys molitrix*) in Tarim River, Xinjiang. *Freshwater Fisheries*, 25-25. (in Chinese)

Xiao H, Zhang Y, Huang D, Polaszek A. 2004. A revision of Homoporus (Hymenoptera: Pteromalidae) of China. *Raffles Bulletin of Zoology*, **52**, 59-66.

Xiong Y. 2009. Dynamics and management of *Pseudaulacaspis pentagona* in Aksu, Xinjiang. *Xinjiang Agricultural Science and Technology*, 54-54. (in Chinese)

Xu J, Jiang H, Zhang R, Aliya, Guo, J. 2012. Growth and patterns of population decline in *Cydia pomonella* adults in Gansu, Xinjiang and Inner Mongolia. *Chinese Journal of Applied Entomology*, **49**, 89-95. (in Chinese, with English abstract)

Yang G. 2005. Harm of introducing the western honeybee *Apis mellifera* L. to the Chinese honeybee *Apis cerana* F. and its ecological impact. *Acta Entomologica Sinica*, **48**, 401-406.

Yang H, Cui Y, Zhang S, Sun X. 2010. The Occurrence and damage of the exotic invasive pest: Western Flower Thrip (*Frankliniella occidentalis*) in Xinjiang. *Xinjiang Agricultural Sciences*, **11**, 024. (in Chinese)

Yang S, Li H, Xu B, Tuerxunnayi T, Tayier A, Zhu X. 2008. Study on species, field identification and biological characters of scale insects in orchards in Tarim Basin. *Xinjiang Agricultural Sciences*, **45**, 276-281. (in Chinese)

Yu G. 1985. Bullfrogs in Xinjiang. *Xinjiang Farm Research of Science and Technology*, **3**, 030. (in Chinese)

Yu J, Qu L. 2001. Occurrence, dynamics and management of *Zygina salina* Mit. in Xinjiang. *Maize Sciences*, **9**, 79-81. (in Chinese)

Yu J, Chen W, Xu Y, Liu X. 2008. Bionomics and control of woolly Apple Aphid (*Eriosoma lanigerum*) in Ili River Valley. *Xinjiang Agricultural Sciences*, **2**, 023. (in Chinese)

Yue C, Zhao B, Wang Y, Zhang J, Zhang X, Wang C. 2013. Risk analysis of *Eulecanium gigantea* in Xinjiang. *Journal of Zhejiang Agricultural and Forestry University*, **30** (in Chinese)

Zhang C, Huang W, Jiang S, Ren D, Zhou N. 2003. Study on bilological characteristics of *Parlatoria oleae* (colvee). *Xinjiang Agricultural Sciences*, **41**, 303-305. (in Chinese)

Zhang L, Miao Y. 2013. Avian community diversity in Urumqi downtown parks and green Lands. *Sichuan Journal of Zoology,* **32**, 97-102. (in Chinese)

Zhang N, Liu C, Wu Y, Zhao L. 2011. Cold hardiness of Hypera postica. *Pratacultural Science*, **28**, 459-463. (in Chinese)

Zhang R. 2012. *Cydia pomonella* (L.). *Chinese Journal of Applied Entomology*, **49**, 26-26. (in Chinese, with English abstract)

Zhang R, Wang X, Shataer A. 2008. Identification and precaution of the ber fruit fly, *Carpomya vesuviana*, a quarantine pest insect in China. *Chinese Bulletin of Entomology*, **44**, 928-930. (in Chinese, with English abstract)

Zhang R, Zhang J, Geng S, Qiu Z. 1998. Niches of wheat aphids in Xinjiang Uygur Autonomous Region. *Acta Entomologica Sinica*, **42**, 40-44.

Zhang R, Xu J, Du L, Wang F, Liu H, Xiang Y, Li X. 2011. Dispersal and impacts of *Cydia pomonella* in China. In: *Annual academic meeting of China Society of Plant Protection*, Suzhou, China. (in Chinese)

Zhang R, Wang F, Zhang T, Chen H, Luo J, Wang Q, Liu W, Pu C, Yan Y. 2012. Progress on monitoring and control of the codling moth, *Cydia pomonella* (L.). *Chinese Journal of Applied Entomology*, **49**, 37-42. (in Chinese, with English abstract)

Zhu G, Hou J, Wang P. 2000. A new pest for quarantine- *Opogona sacchari* introduced into Xinjiang with ornamental plants. *Xinjiang Agricultural Science and Technology*, 185. (in Chinese)
